# A Noninvasive Isotopic Approach to Estimate the Bone Lead Contribution to Blood in Children: Implications for Assessing the Efficacy of Lead Abatement

**DOI:** 10.1289/ehp.7241

**Published:** 2004-10-07

**Authors:** Roberto Gwiazda, Carla Campbell, Donald Smith

**Affiliations:** ^1^Environmental Toxicology, University of California, Santa Cruz, California, USA; ^2^The Children’s Hospital of Philadelphia, Philadelphia, Pennsylvania, USA

**Keywords:** abatement efficacy, blood lead, bone lead, fecal lead, lead abatement, lead hazards, lead isotopes, lead loadings

## Abstract

Lead hazard control measures to reduce children’s exposure to household lead sources often result in only limited reductions in blood lead levels. This may be due to incomplete remediation of lead sources and/or to the remobilization of lead stores from bone, which may act as an endogenous lead source that buffers reductions in blood lead levels. Here we present a noninvasive isotopic approach to estimate the magnitude of the bone lead contribution to blood in children following household lead remediation. In this approach, lead isotopic ratios of a child’s blood and 5-day fecal samples are determined before and after a household intervention aimed at reducing the child’s lead intake. The bone lead contribution to blood is estimated from a system of mass balance equations of lead concentrations and isotopic compositions in blood at the different times of sample collection. The utility of this method is illustrated with three cases of children with blood lead levels in the range of 18–29 μg/dL. In all three cases, the release of lead from bone supported a substantial fraction of the measured blood lead level postintervention, up to 96% in one case. In general, the lead isotopic compositions of feces matched or were within the range of the lead isotopic compositions of the household dusts with lead loadings exceeding U.S. Environmental Protection Agency action levels. This isotopic agreement underscores the utility of lead isotopic measurements of feces to identify household sources of lead exposure. Results from this limited number of cases support the hypothesis that the release of bone lead into blood may substantially buffer the decrease in blood lead levels expected from the reduction in lead intake.

Most (> 70%) of the body lead burden in children is contained within the skeleton (Barry 1981). Because lead is qualitatively a biologic analog to calcium, its uptake and release from the skeleton are partly controlled by processes affecting bone growth and turnover (Hu et al. 1998; O’Flaherty 1998). In adults, skeletal lead is contained within long-lived compartments of cortical [elimination half-life (*t*_1/2_) > 5–10 years] and trabecular (elimination *t*_1/2_ > 1 year) bone, with comparatively small amounts of lead in tissue compartments that rapidly exchange with extracellular fluid and plasma (Hu et al. 1998; Leggett 1993; O’Flaherty 1998; Rabinowitz et al. 1976). In children, however, the turnover rates of skeletal reservoirs of lead and the impact of bone lead releases on blood lead content are not well understood. In children exposed to lead hazards, the accumulation of lead in bone and other tissues is of serious concern because these body lead stores are believed to serve as internal sources of lead to blood during bone remodeling (Gulson et al. 1996, 1997b; Leggett 1993; Nordberg et al. 1991; O’Flaherty 1994). Moreover, mobilization of accumulated skeletal lead stores back into blood is suspected to be responsible for the apparent limited success of various lead hazard control measures to decrease blood lead levels in exposed children (Burgoon et al. 1995; Rust et al. 1999). These abatement efforts typically result in reductions of blood lead levels in exposed children of no more than 30% when evaluated within several months after intervention [U.S. Environmental Protection Agency (EPA) 1995].

The importance of bone lead storage and mobilization in controlling blood lead levels has been documented in adults (Rabinowitz et al. 1976; Smith et al. 1996). Increased bone resorption in winter months (Oliveira et al. 2002), during pregnancy and lactation (Gulson et al. 1997a; Lagerkvist et al. 1996; Manton et al. 2003; Rothenberg et al. 2001; Silbergeld 1991; Tellez-Rojo et al. 2002), under hyperthyroidism conditions (Goldman et al. 1994), or due to skeletal disease (Berlin et al. 1995) has been associated with elevated blood lead levels. In addition, experimental lead isotope studies in nonhuman primates have demonstrated lead releases from bone to blood (Inskip et al. 1996). The existence of a relationship between bone remodeling and blood lead content has been also hypothesized for children (Angle et al. 1995; Gulson et al. 1996; Gwiazda and Smith 2000; Hu et al. 1998; Manton et al. 2000; O’Flaherty 1994; Rust et al. 1999), though this link is difficult to document partly because of the challenges of determining bone lead burdens in pediatric populations (Hu et al. 1998).

Measurements of bone lead content in children could be used to establish empirical relationships between bone and blood lead levels in pediatric populations. However, this relationship would be affected by other factors such as current lead intake, age, and history of exposure that are thought to affect the nature of the bone lead–blood lead relationship. Nevertheless, because of the importance of bone lead in human lead toxicokinetics, the potential effect of bone turnover on blood lead content has been included in the structure of pharmacokinetic models of childhood lead poisoning. These include the Integrated Exposure and Uptake Biokinetic model (White et al. 1998), O’Flaherty’s (1998) physiologically based pharmacokinetic model, and Leggett’s (1993) biokinetic lead model. Validation of these models for bone lead release has been limited, however, because of the scarcity of suitable pediatric data for accurate ground-truthing and calibration.

Lead isotopic methods provide an alternative approach to estimate the impact of endogenous sources of lead on blood lead content (Gulson et al. 1997b; Smith et al. 1996). In its simplest form, this approach apportions the blood lead isotopic composition as a mixture of two end members: the lead isotopic composition of intake and the lead isotopic composition of the endogenous source(s). The critical challenge in applying this approach is the characterization of the isotopic composition of the end members contributing lead to blood (e.g., external sources and the skeleton). To this end, a variety of experimental designs have been used in adults. In these designs, the isotopic composition of the lead intake either was estimated from analysis of environmental samples (Gulson et al. 1995, 1996; Manton 1985; Smith et al. 1996) and from duplicate diets (Gulson et al. 1997a; Manton 1985; Rabinowitz et al. 1976) or was purposefully changed (Facchetti 1989; Rabinowitz et al. 1973, 1976, 1977). The lead isotopic composition of the endogenous skeletal source was estimated on the basis of the assumed historical exposure (Gulson et al. 1995, 1997b) or measured directly on bone samples (Manton 1985; Smith et al. 1996). In some cases the fraction of lead derived from the skeleton was calculated from simple proportionality (Gulson et al. 1995; Smith et al. 1996), whereas in others its computation required the use of mathematical models (Colombo and Fantechi 1983; Rabinowitz et al. 1973, 1976, 1977).

Studies by Gulson et al. (1996, 1997b), who used the lead isotopic approach to estimate the skeletal lead contribution to blood in children, took advantage of the fact that the studied children had lived at a younger age in locations with a presumably well-characterized environmental lead isotopic composition, which was different from that of their current exposure. The authors then assumed that the skeletal lead carried a homogeneous isotopic signature from the earlier exposure. Although their work provides supporting evidence for the contribution of bone lead to blood lead in children, their methodologic approach is limited to the special circumstances of a child moving to a very different location. In most cases, however, the lead isotopic composition of children’s skeletal tissue cannot be empirically ascertained, and therefore the apportionment of the blood lead isotopic composition between the skeletal and the exogenous end members is not possible. Isotopic measurements of shed deciduous lead in teeth could serve as proxy of the lead isotopic composition of bone, but this opportunistic sampling would be useful only for children ≥ 5 years of age. Similarly, it is often difficult to obtain a weighted average lead isotopic composition of lead intake, given the various possible sources and pathways of exposure (dust, soil, food, air) from which children absorb lead. Isotopic characterization of lead intake has been done from duplicate diets. However, this sampling method does not include all sources of lead exposure to the child, especially for younger children who may ingest high amounts of lead from environmental sources through increased hand-to-mouth activity.

Here we present a noninvasive isotopic approach to estimate the magnitude of the bone lead contribution to blood in children following household lead remediation. This approach does not require lead isotopic measurements of bone, nor does it assume the lead isotopic ratio of bone on the basis of the child’s lead exposure history. Instead, blood and feces are sampled for lead concentration and isotopic analyses before and after implementation of environmental lead hazard control measures to reduce the child’s lead exposure(s). Estimation of the bone lead contribution to blood using this method is illustrated with three cases of childhood lead poisoning. In addition, the sources of lead exposure to the child are identified from a comparison of the lead isotopic compositions of household sources and feces, using the latter as a surrogate measure of the magnitude and isotopic composition of lead intake.

## Materials and Methods

### Experimental approach.

The isotopic composition of blood is a function of the isotopic compositions and relative lead contributions of exogenous intake and endogenous sources. Here, we assume that the isotopic composition of feces reflects the isotopic composition of lead intake. However, the isotopic composition of bone is not known. To calculate this value and be able to solve the relative lead contributions from intake and from the skeleton to blood, we rely on an induced change in the magnitude and isotopic composition of lead intake through the elimination of identified household sources of lead exposure (i.e., household lead abatement intervention). The assumption in this approach is that by reducing the magnitude of the child’s lead intake, the relative contribution of lead to blood from the endogenous skeletal source increases and the lead isotopic composition of blood shifts toward the isotopic value of bone.

We applied a system of linear equations to calculate the endogenous lead contribution to blood, with lead content and isotopic composition of blood and feces before and after intervention as independent variables. More generally, this system of equations can be applied between any two time points with different blood lead levels, regardless of the cause and direction of change in blood lead content (increase or decrease).

We used the following mass balance equations for lead content and for lead isotopes at two different time points, *t*_1_ and *t*_2_, to describe the mixing of lead in blood:


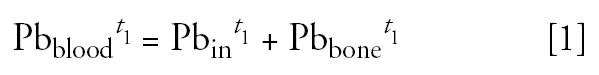



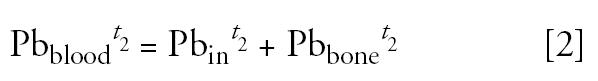



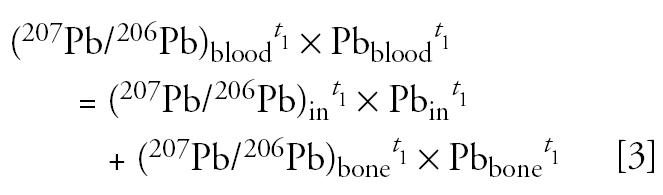



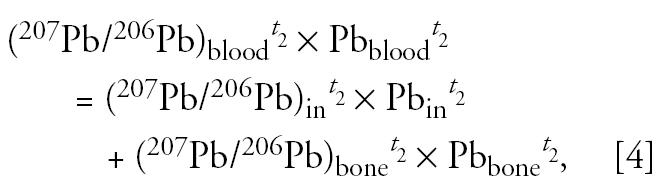


where Pb_blood_ is the concentration of lead in blood, in micrograms per deciliter. Pb_in_ and Pb_bone_ are the amounts of lead in blood from external intake and from bone, respectively, in micrograms per deciliter. (^207^Pb/^206^Pb)_in_ is the isotopic ratio of lead in blood derived from external intake, and it is assumed to be identical to the isotopic composition of lead measured in feces, as follows:


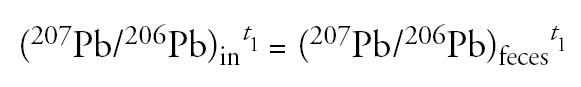



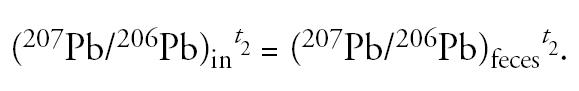


It is also assumed that the lead isotopic composition of bone did not change between the two time points considered, as follows:


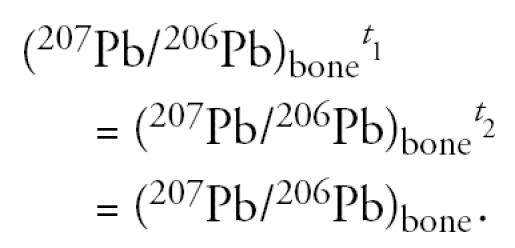


It is assumed that the amount of lead in blood from the external intake, Pb_in_, is proportional to the rate of lead intake. Here, the rate of lead excretion (Pb_feces_, in micrograms lead per day) is used as a surrogate of the rate of lead intake, as follows:


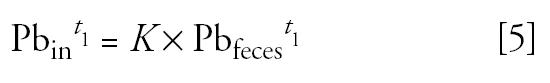



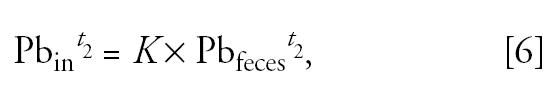


where *K* is a biokinetic constant (in micrograms per deciliter/micrograms excreted per day) that relates the lead content of feces to the amount of lead in blood from external intake. This biokinetic constant *K* is different from the more familiar term biokinetic slope factor (BKSF), which refers to the increase in blood lead per unit of lead absorbed (instead of excreted) across the gastrointestinal (GI) tract (Bowers et al. 1994; Bowers and Cohen 1998; U.S. EPA 2003). This approach yields a solvable system of six equations with six unknowns, *K*, Pb_in_*^t^*^_1_^, Pb_in_*^t^*^_2_^, Pb_bone_*^t^*^_1_^, Pb_bone_*^t^*^_2_^, (^207^Pb/^206^Pb)_bone_, with no need for parameterization (e.g., use of independently obtained parameters that describe fractional lead absorption across the GI tract, rate of bone turnover, etc.).

Notably, this method can be applied to obtain the relative contributions of lead from the intake and the skeleton to blood only when the lead isotopic compositions of the external (intake) and internal (skeleton) sources are different. In practical terms, because the isotopic composition of the skeletal lead source typically cannot be known, this approach can be applied only if the difference between the blood and intake lead isotopic compositions is greater than the isotopic measurement error.

### Subjects.

Children were recruited by the Children’s Hospital of Philadelphia from referrals by the Philadelphia Childhood Lead Poisoning Prevention Program. Inclusion criteria were that blood lead level was between 15 and 35 μg/dL, the child was < 6 years of age, the child spent most of his or her waking time within a single household environment, and the blood and fecal lead isotopic compositions were measurably different (to confirm the latter criterion, blood and fecal samples were analyzed within 2 weeks following recruitment). Four cases were recruited. Three boys, 14, 20, and 46 months of age, met the inclusion criteria and were retained in the study (Table 1). One case did not meet the latter criterion above and was excluded from follow-up. Informed written consent was obtained from all parents/guardians. All procedures used in the recruitment of subjects, including the administered questionnaire and the collection of biologic and environmental samples, received prior review and approval by the human subjects institutional review boards of the University of California at Santa Cruz and the Children’s Hospital in Philadelphia.

### Sample collection.

Blood, 5-day complete fecal samples, and household environmental samples were collected at enrollment. Within 2 months, a state-certified lead abatement contractor conducted a thorough cleaning of the household environment, including high-efficiency particulate air (HEPA) vacuuming and wet washing of all horizontal surfaces with trisodium phosphate detergent. Blood and 5-day fecal samples were collected a second time (*t*_2_) at least 1 month after the house-hold cleaning. Finally, a third round of blood and 5-day fecal collections was performed at least 3 months after the second round of sample collection (Table 1).

Blood samples (3 mL) were collected into low-lead heparinized Vacutainer tubes (no. 367734; Becton-Dickinson, Franking Lakes, NJ) by the Children’s Hospital of Philadelphia. Parents collected daily fecal samples in diapers provided by the study (LUVS Ultra Leak Guards no. 4; Procter & Gamble, Cincinnati, OH) or in perforated urine collection “hats” (McKesson Medical Surgical, Richmond, VA) that had been prewashed with distilled water and air-dried in a filtered-air environment. Household samples of all deteriorated paints, floor dusts, and soils, if appropriate, were sampled by the Philadelphia Health Department following U.S. Department of Housing and Urban Development (HUD) guidelines (HUD 1995). All samples were shipped to the University of California at Santa Cruz for lead concentration and isotopic composition analyses, as described below.

### Analytical techniques.

Processing of biologic samples was conducted under trace-metal–clean HEPA-filtered air (Class-100) conditions using clean techniques (Smith et al. 1992). Acids used in sample processing and analyses were quartz double distilled, water was ultrapure grade (18 MΩ.cm), and all sample-processing plasticware was acid cleaned (Flegal and Smith 1992).

Blood samples were processed in triplicate as described in Gwiazda and Smith (2000). Ultrapure water was added to fecal samples (at least two parts water to one part feces, wt/wt), and the mix was homogenized with a stainless steel blender. Duplicate aliquots (~2.5 g each) of the fecal homogenate were dried and digested overnight in 2–3 mL sub-boiling 16N HNO_3_. After evaporation to dryness, samples were reconstituted in 1N HNO_3_, and centrifuged at 15,000 × *g*. The supernatant was spiked with ^209^Bi for analysis in an inductively coupled plasma mass spectrometer (ICP-MS), as described below.

Paint (0.1–0.2 g) and soil (~1 g) samples were homogenized with mortar and pestle, weighed, and digested in trace-metal–grade 16N HNO_3_ for at least 12 hr. After evaporation to dryness, samples were reconstituted in 1N HNO_3_, filtered (Whatman filter paper no. 4; Fisher Scientific, Pittsburgh, PA), and spiked with ^209^Bi for analysis by ICP-MS. Dust wipes were digested in a similar fashion.

A double focusing magnetic sector ICP-MS (Finnigan Element, Thermo Electron Corporation, Bremen, Germany) was used for lead isotopic and concentration measurements using the method of Gwiazda et al. (1998), but with shorter scan times of 10 msec for each mass. ^204^Pb abundance was not measured. National Institute of Standards and Technology (NIST) standard reference material (SRM) 955b level 4 (lead in blood) was used to evaluate the precision of lead isotopic and accuracy of lead concentration measurements in blood. The measured lead concentration of the 955b blood SRM was 38.6 ± 1.3 μg/dL (2× SE, *n* = 5), in good agreement with the certified value of 39.4 μg/dL. The precision of the blood ^207^Pb/^206^Pb ratio measurements over the course of the study was 0.2% [2× relative standard deviation (RSD)], based on the analyses of NIST 955b blood SRM over 5 different days of analyses. The precision of blood ^207^Pb/^206^Pb and ^208^Pb/^206^Pb ratio measurements within an analytical run was 0.16 and 0.26% (2× RSD), respectively, based on triplicate analyses of the children’s blood samples at each single collection interval. The average difference in ^207^Pb/^206^Pb and ^208^Pb/^206^Pb ratios between homogenized feces duplicates was 0.11 and 0.16%, respectively (*n* = 39 pairs). Precision and accuracy of lead isotopic ratios of environmental samples were estimated from repeated measurements of NIST 981 (common lead isotopic standard reference material). The long-term precision of NIST 981 ^207^Pb/^206^Pb and ^208^Pb/^206^Pb ratios was 0.13 and 0.10% (2× RSD, *n* = 5 different measurement days), and the accuracy was within 0.05% of the certified ratio values.

Diaper blank was estimated to be approximately 5.8 ng lead per diaper, based on the analyses of ultrapure water rinsed over the inner surface of new diapers (*n* = 12). Fecal sample contamination associated with homogenization was < 5 ng lead, based on total procedural homogenization blanks (*n* = 6) processed with each batch of feces. These lead blank values are three orders of magnitude less than the typical amount of lead found in feces in a diaper, indicating that fecal lead contamination associated with collection and processing was negligible.

## Results

### Relationships between lead isotopic ratios of environmental, feces, and blood samples.

**Figure 1:**
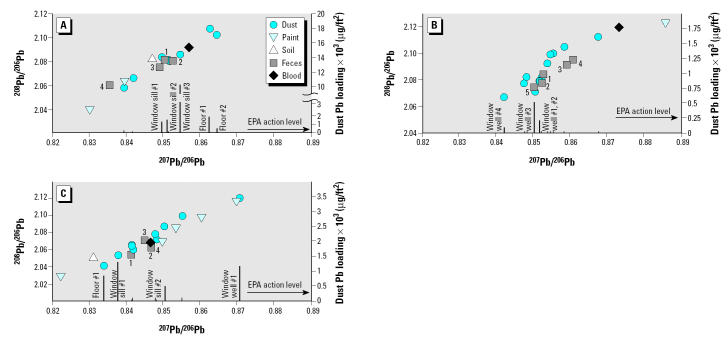
Lead isotopic ratios of blood, feces, and environmental samples, and household dust lead loadings from the first visit (*t*_1_). The vertical bar below each dust isotopic composition symbol is the lead loading of that particular dust sample according to the right ordinate scale. Numbers adjacent to the feces symbols indicate the day of the 4–5 day sequential feces sample collection. (*A*) Case 1. Fecal lead content (μg): day 1, 7.0; day 2, 6.6; day 3, 2.9; day 4, 9.0. (*B*) Case 2. Fecal lead content (μg): day 1, 5.1; day 2, 6.0; day 3, 4.7; day 4, 1.9; day 5, 7.0. (*C*) Case 3. Fecal lead content (μg): day 1, 4.7; day 2, 41; day 3, 240; day 4, 7.3.

In this study we were able to distinguish analytically the various household sources of environmental lead, because the overall range of ^207^Pb/^206^Pb ratios of environmental samples from all households (i.e., 4%) was approximately 20 times larger than the isotope ratio measurement error (< 0.2%) (Figure 1). In general, the lead isotopic compositions of feces from the first round of sampling (i.e., before household intervention) match (cases 1 and 2, Figure 1A,B) or are bracketed (case 3, Figure 1C) by the lead isotopic compositions of the household dusts with lead loadings exceeding U.S. EPA action levels that were collected in the same visit.

**Figure 2:**
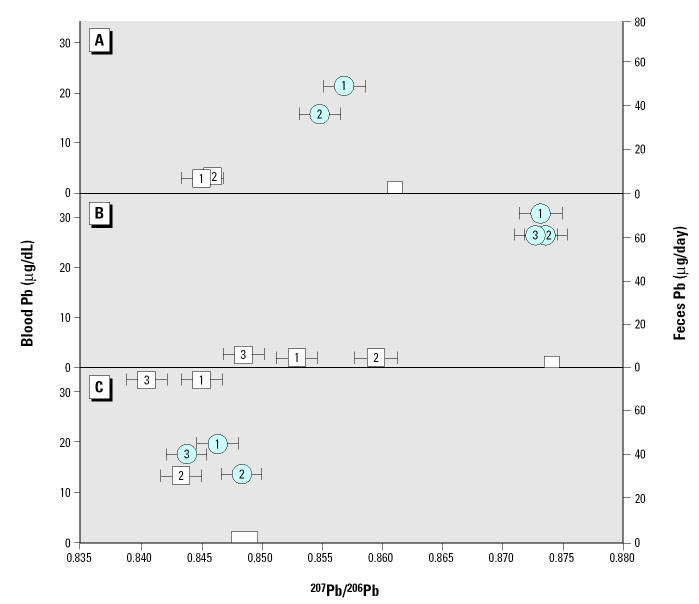
Lead content and isotopic composition of blood (circles) and feces (squares) from cases 1, 2, and 3 (*A*, *B*, and *C*, respectively). The visit number (1, 2, or 3) is shown on the symbols. The fecal lead isotopic composition shown is the lead content–weighted average of the lead isotopic compositions of the daily feces collected in the visit. The white rectangles on the x-axes are the ranges in calculated isotopic composition of lead in the skeleton.

The lead content of feces of case 3 in the first visit indicates very variable daily lead intake (Figure 1C). The highest daily fecal lead content, 240 μg Pb, is up to 40 times higher than the average daily fecal lead content of approximately 6 μg Pb/day of the children in the other two cases. We calculated a lead content–weighted grand average fecal isotopic composition for each sampling round based on the lead content and isotopic composition of the daily fecal samples (Figure 2). These calculations indicate that the isotopic compositions of blood and average feces of case 3 (Figure 2C) from the first sampling round (*t*_1_) are in much closer proximity to each other than what is observed in the other two cases, consistent with a greater relative impact of recent lead exposures on blood lead levels.

In contrast to case 3, the lead intakes of cases 1 and 2 in the first round of sampling (*t*_1_) were low, as reflected in the lead content of feces (~ 6.5 and 5 μg/day, respectively) (Figure 1A,B). However, the blood lead levels in these two cases (20.3 and 29.3 μg/dL, respectively) are comparable with or higher than in case 3 (18.3 μg/dL). In addition, in both cases 1 and 2, blood from all sampling rounds contained higher ^207^Pb/^206^Pb ratios than the average feces (Figure 2A,B).

Blood lead levels declined between the first (*t*_1_) and second (*t*_2_) visits in all three cases, even though in cases 1 and 2 there was no significant change in the fecal lead content (Figure 2A,B). In these two cases the blood isotopic composition at the second visit (*t*_2_) moved toward the isotopic composition of feces (case 1) or remained unchanged (*t*_2_, case 2).

In contrast to this, in case 3 the fecal lead content decreased postintervention (*t*_2_) and the blood ^207^Pb/^206^Pb ratios shifted away from the isotopic composition of feces (Figure 2C). When the lead content of feces again increased at the final round of sampling (case 3, *t*_3_), the blood lead level also increased (from 12.9 to 16.6 μg/dL at *t*_2_ and *t*_3_, respectively), and its isotopic composition shifted back closer to the fecal lead ^207^Pb/^206^Pb ratio (Figure 2C). No significant change was observed in case 2 between *t*_2_ and *t*_3_.

### Estimates of bone lead contribution to blood.

Estimates of the amount of lead in blood from bone at each collection time point are obtained by applying Equations 1–6 to blood and fecal lead concentrations and isotopic ratios of any two sampling rounds (i.e., *t*_1_ and *t*_2_, *t*_1_ and *t*_3_, *t*_2_ and *t*_3_). Thus, in cases 2 and 3, where three sampling rounds took place, it is possible to obtain two estimates of the bone lead contribution to blood for each collection time point. This is because each sampling round is used in two different pairs of time points in the calculations. For example, two estimates of bone lead contribution to blood were calculated for *t*_2_, one estimate based on the *t*_1_ and *t*_2_ sample collection pair, and the other based on the *t*_2_ and *t*_3_ sample collection pair (Table 2).

The estimated bone lead contribution to blood in the oldest child (46 months of age, case 2) is consistent throughout the three sampling rounds and amounts to > 90% of blood lead. In other words, uptake of lead from external sources in that child supported < 10% of the lead in blood throughout the 7.4 months encompassed by the sampling rounds. Because blood lead levels in that child ranged between approximately 25 and 29 μg/dL throughout the study, these results indicate that the chronically elevated blood lead levels may be attributed to the mobilization of substantial bone lead stores. In case 1, where only two sampling visits took place, the estimated bone lead contribution averaged approximately 65% but decreased slightly from the first visit (73%) to the second visit (58%), consistent with the reduction in blood lead levels (from 20.3 to 14.9 μg/dL) and with the absence of a reduction in fecal lead elimination (i.e., lead intake) over the time interval (Tables 1 and 2, Figure 2A).

In case 3, the amount of lead in blood from bone is more variable. For this case, the estimates of the bone lead contribution to blood on the basis of the two different sampling pairs are 36% at *t*_1_ (average of 19 and 53% calculated using the *t*_1_–*t*_2_ and *t*_1_–*t*_3_ collection times, respectively), 65% at *t*_2_ (average of 59 and 70% from pairs *t*_1_–*t*_2_ and *t*_2_–*t*_3_, respectively), and 40% at *t*_3_ (average of 33 and 48% from pairs *t*_1_–*t*_3_ and *t*_2_–*t*_3_, respectively). This variability in the estimates of bone lead contribution calculated using two different sampling pairs is produced by the relative size of the analytical measurement error compared with the isotopic differences between blood and feces from two collection time points. When isotopic differences between samples collected at different times are small, the measurement uncertainty in the isotopic values results in large uncertainties in the estimates of all parameters calculated from Equations 1–6.

The biokinetic factor *K* (Equations 5 and 6) relates the amount of lead in blood from external intake with the lead content of feces, that is, with the amount of lead ingested but not absorbed. If it is assumed that GI absorption of lead in infants and small children is on the order of 50% (Alexander et al. 1974; Ziegler et al. 1978), the value of *K* should be numerically equivalent to the more commonly used BKSF defined as the increase in blood lead per unit of lead absorbed across the GI tract (Bowers and Cohen 1998; Bowers et al. 1994; U.S. EPA 2003). Data from infants fed formula mixed with leaded water (Sherlock and Quinn 1986) suggest a BKSF for small children of 0.21–0.11 in the blood range of 13–30 μg/dL, if GI lead absorption is assumed to be 0.5. Calculated biokinetic factors (*K*) (Table 2) range from 0.83 to 0.12, although they are generally much more consistent for a given child. Comparison of biokinetic factors (*K*) across children, and even across studies, should be done with caution because this term is not normalized to body weight.

## Discussion

The three case studies presented here serve to illustrate the application of this noninvasive isotopic approach to estimate the bone lead contribution to blood in lead-poisoned children. These results substantiate that in lead-exposed children reductions in blood lead levels post-intervention may be buffered by the release of significant amounts of lead from bone into blood and thus may not adequately reflect reductions in lead exposure from environmental sources. The endogenous source of this lead mobilized into blood is presumed to be the skeleton, because the skeleton contains most of the body lead burden. Thus, the ability of a household lead abatement intervention to produce considerable reductions in the blood lead level of a chronically lead exposed child may be substantially limited by the large contribution of bone lead to blood.

This is best demonstrated by case 2, the oldest child examined here (46 months of age at enrollment), whose fraction of lead in blood from bone was calculated to be > 90% throughout the study. Supporting this, the lead intake of this child was comparatively low (~ 6 μg/day fecal lead elimination) and constant throughout the three sampling visits, yet the blood lead level was very elevated and decreased only slightly over time (from 29.3 down to 25.2 μg/dL over 7 months). Thus, even if the lead intake had been completely eliminated by the household abatement intervention, the expected decrease in blood lead level would have been very small. This case, in particular, illustrates the limitations of assuming that blood lead levels are direct indicators of current environmental lead exposure and that lead hazard control measures would necessarily be efficacious in significantly reducing blood lead levels.

In cases 1 and 3, the estimated bone lead contribution to blood was calculated to be smaller than in case 2 (i.e., ~ 40–65% vs. > 90%) and, at least in case 3, more variable over time. The different estimated contributions of bone lead to blood lead over time in the three children studied here could be due to a number of factors, including differences in exposure history and levels of lead accumulated in bone. The child with the largest bone lead contribution to blood (case 2, > 90%) was the oldest of the three children and had a very low lead intake, based on fecal lead elimination. In this case, a larger store of bone lead accumulated over a prolonged period (i.e., years) of exposure to elevated environmental lead levels could have maintained elevated blood lead levels that only very slowly decreased over time once the exposures were controlled. Under this scenario, reduction of the elevated environmental exposures to the case 2 child may have occurred before the conduct of this study, consistent with his relatively low fecal lead content at enrollment. In the other two cases of younger children (~ 1.5 years old), the bone lead contribution to blood was smaller and more variable (at least in case 3), suggesting a smaller reservoir of lead in bone, possibly due to a shorter history of environmental exposure.

Historically, the efficacy of lead abatement practices for reducing childhood lead exposures has been evaluated based on reductions in blood lead levels as indicators of lead exposure/uptake (Aschengrau et al. 1994, 1998; CDC 1991; Charney et al. 1983; Farfel and Chisolm 1990; Hilts et al. 1995; Kimbrough et al. 1994). This approach does not sufficiently consider the very important contribution of accumulated bone lead stores in “buffering” reductions in blood lead levels postintervention. Although it may be more difficult to verify, a more accurate appraisal of the effectiveness of lead hazard control measures would be based on their success in reducing lead intake.

Fecal lead content measured over several days is one possible approach to estimating the overall magnitude of childhood lead intake. Fecal lead content should give an integrated measure of lead exposure/intake from all sources, dietary and environmental, inside and outside the home. In contrast, other approaches such as duplicate diet sampling may not sufficiently reflect total lead exposure/intake because duplicate diets do not reflect potentially important environmental sources of lead to children living in older housing or in the proximity of soils with high lead content. Similarly, hand wipes may provide an estimate of nondietary environmental lead exposure in some cases (Duggan et al. 1985), although they do not reliably reflect ingestion of environmental lead.

There are, however, limitations with the use of fecal lead content as a measure of lead intake. First, collection of complete fecal samples over multiple days may not be feasible in some cases. Second, variability among children in GI lead absorption should ideally be taken into consideration if fecal lead content were to be used as a direct surrogate of lead uptake and intake. Third, because excreted fecal lead reflects unabsorbed ingested lead in addition to lead eliminated via endogenous fecal (e.g., biliary) routes, variation in these physiologic processes from child to child may introduce variation not attributable to environmental lead exposure. Nonetheless, fecal lead content still may be the among the most accurate indicators of the amount and isotopic composition of lead the child is ingesting, and as a result, it may serve as a useful quantitative index of the extent of oral lead exposure from all sources (diet and environment).

### Assumptions and limitations of this isotopic approach.

The mathematical approach used here to calculate the bone lead contribution to blood relies on a number of assumptions, including *a*) that the lead isotopic composition of feces reflects the lead isotopic composition of ingested lead that is incorporated into the circulation, and *b*) that the isotopic composition of the skeleton remains constant throughout the 3- to 6-month interval between consecutive sampling visits. Although prior studies have not systematically validated the first assumption, it is supported by a number of published observations. Studies where lead intake and excretion were measured in animals and humans showed that an increase in lead intake is quickly followed by an increase in fecal lead excretion (Barltrop and Killala 1967; Kehoe 1987; Ziegler et al. 1978). In addition, fecal lead excretions by children suffering elevated lead exposures have been shown to correlate with the degree of lead paint hazards in their household environment (Hammond et al. 1980). Perhaps most relevant to the present study, Rabinowitz (1987) showed that the lead isotopic compositions of leaded paints in the household environments of lead-poisoned children matched the isotopic compositions of the children’s bloods and their excreted feces. These latter observations are replicated in the three cases reported here where the lead isotopic compositions of feces matched or were within the range of the lead isotopic compositions of the household dusts with the highest lead loadings.

An additional factor related to the first assumption that is not included in the model but that may affect the utility of fecal lead content as a surrogate of lead intake is the elimination of lead into feces via endogenous (e.g., biliary) pathways. Biliary lead excretion has been shown to range between 40 and 85% of total body lead excretion in nonhuman primates (Cremin et al. 2001; O’Flaherty et al. 1996) and < 46% in adult humans (Rabinowitz et al. 1976), and it is estimated to be at least 50% in infants (Ziegler et al. 1978). Assuming that the lead isotopic composition of bile matches that of blood, the biliary elimination of endogenous lead into feces would shift the lead isotopic value of feces toward the isotopic value of blood, although the extent of this shift would depend on the amount (e.g., micrograms) of lead eliminated from this biliary route compared with the amount of lead in the feces (i.e., unabsorbed lead from oral intake). Consequently, the measured difference in lead isotopic composition between feces and blood would be smaller than the true isotopic difference between blood and the oral intake. Here, we conservatively chose to not include in the model a term for endogenous biliary lead excretion into feces because endogenous fecal lead excretion is unknowable for a particular child, and including this term in the model would introduce a large uncertainty in the calculation of the bone lead contribution to blood. Functionally, the impact of not including this variable in the model produces calculated bone lead contributions to blood that, if anything, are minimum values that could be larger for a particular child.

The second assumption of this model is that the isotopic composition of the skeleton remains constant throughout the sampling visits [i.e., the model assumes (^207^Pb/^206^Pb)_bone_*^t^*^_1_^ = (^207^Pb/^206^Pb)_bone_*^t^*^_2_^]. It is possible, however, that changes in the blood isotopic composition because of reductions in lead intake after intervention, for example, could produce small changes in the isotopic composition of metabolically active regions of bone that exchange lead with blood. Accordingly, the bone lead isotopic composition could change slightly toward that of blood, rather than remain constant throughout the study. If allowance is made in the model for a change in bone isotopic composition toward that of blood, as the magnitude of external sources of exposure changes, the estimated contribution of lead from bone to blood actually increases. Thus, the approach used here, which assumes that the bone lead isotopic composition does not change with time, yields a minimum calculated value for the contribution of bone lead to blood.

## Conclusions

We present a noninvasive isotopic approach to estimate the bone lead contribution to blood in children following interventions to reduce environmental lead exposures. Illustration of this method using three cases of lead-poisoned children provides evidence that mobilized skeletal lead stores may contribute a significant fraction of lead to blood, up to 90% or more in one case presented here, which may substantially “buffer” reductions in blood lead levels after environmental lead remediation. Because the accumulated skeletal lead burden likely varies from child to child, depending partly on the child’s age and lead exposure history, it should be expected that blood lead levels would decrease at different rates postintervention, depending on the contributions of bone lead to blood in different children. This suggests that the efficacy of lead hazard remediation efforts should be evaluated over prolonged periods (i.e., ≥ 6–12 months) to allow adequate time for depletion of accumulated skeletal lead stores and a reduction in their absolute contribution to blood lead levels. Observations from this study also support the use of fecal lead content and isotopic composition as a proxy for the identification of sources of lead exposure.

## Figures and Tables

**Table 1:** Age (months) and blood lead levels (BPb; μg/dL) of children at enrollment, household lead abatement, and subsequent sample collection visits.

	Enrollment (*t*_1_)			2nd collection (*t*_2_)		3rd collection (*t*_3_)	
Case	Age	BPb	Abatement age	Age	BPb	Age	BPb
1	14	20.3	15.2	16.1	4.9	Withdrew*^a^*	
2	46	29.3	47	49	25.4	53.4	25.2
3	20	18.3	22.1	27	12.9	36	16.6

a Child withdrew from the study before the third visit.

**Table 2:** Calculated fraction of lead in blood from bone, ^207^Pb/^206^Pb of bone, and biokinetic factor [in (μg/dL)/(μg/day)], from Equations 1–6.

	Sampling round		
Case	*t*_1_	*t*_2_	*t*_3_
1			
Fraction of lead in blood from bone (%)	73	58	NA
Bone lead isotopic composition	0.8610	0.8610	
Biokinetic factor *K* (Equation 5)	0.83	0.83	
2*^a^*			
Fraction of lead in blood from bone (%)	91–92	91–96	92–96
Bone lead isotopic composition	0.8737–0.8750	0.8742–0.8750	0.8737–0.8742
Biokinetic factor *K* (Equation 5)	0.48–0.14	0.21–0.48	0.14–0.21
3*^a^*			
Fraction of lead in blood from bone (%)	19–53	59–70	33–48
Bone lead isotopic composition	0.8474–0.8517	0.8505–0.8517	0.8474–0.8505
Biokinetic factor *K* (Equation 5)	0.12–0.21	0.15–0.21	0.12–0.15

NA, not applicable.

a In cases 2 and 3, two values can be calculated for each time point by pairing data from each sample collection time with data from either one of the other two collection times (see “Materials and Methods”). For example, for *t*_1_ Equations 1–6 can be applied in combination with *t*_2_ or in combination with *t*_3_.
